# Pancreatic Stiffness in Patients with Type 2 Diabetes Mellitus Assessed by 2-Dimensional Shear Wave Elastography: A Pilot Case–Control Study

**DOI:** 10.5152/tjg.2026.25617

**Published:** 2026-08-17

**Authors:** Yahya Erdem İnce, Orhan Sezgin, Serkan Yaraş, Osman Özdoğan, Ramazan Gen

**Affiliations:** 1 Division of Endocrinology, Department of Internal Medicine, Çukurova University Faculty of Medicine, Adana, Türkiye; 2 Division of Gastroenterology, Department of Internal Medicine, Mersin University Faculty of Medicine, Mersin, Türkiye; 3 Division of Endocrinology, Department of Internal Medicine, Mersin University Faculty of Medicine, Mersin, Türkiye

**Keywords:** Diabetes, elastography, pancreatic stiffness, steatosis

## Abstract

**Background/Aims:**

Shear wave elastography (SWE) is a noninvasive method for assessing tissue stiffness. This study evaluated pancreatic stiffness in patients with type 2 diabetes mellitus (T2DM) and examined its association with metabolic factors and microvascular complications.

**Materials and Methods:**

This prospective case–control study included 45 patients with T2DM and 45 healthy controls. Pancreatic stiffness was measured using 2-dimensional SWE (2D-SWE). Clinical, anthropometric, and laboratory data were collected, and diabetic microvascular complications were assessed.

**Results:**

A total of 90 participants were included, comprising 45 patients with T2DM and 45 healthy controls. Pancreatic stiffness was significantly higher in patients with T2DM than in controls across all pancreatic regions (*P* < .001). However, pancreatic stiffness did not differ significantly between patients with T2DM with and without microvascular complications (*P* > .05). Pancreatic steatosis (PS) was more prevalent in patients with T2DM than in healthy controls (*P* = .001). Among patients with T2DM, pancreatic stiffness was significantly higher in those with steatosis than in those without (*P* = .035), and stiffness increased with the severity of steatosis (*P* = .021). Body mass index and waist circumference (WC) were independently associated with increased pancreatic stiffness, with WC accounting for a significant proportion of the variance (*P* < .001).

**Conclusion:**

Pancreatic stiffness was significantly higher in patients with T2DM than in healthy controls and was primarily associated with metabolic factors, particularly obesity and PS, rather than microvascular complications. These findings suggest that increased pancreatic stiffness may reflect metabolic associated pancreatic alterations.

**Main Points**

- Pancreatic stiffness is significantly higher in patients with type 2 diabetes than in healthy controls.
- Increased pancreatic stiffness is associated with metabolic factors (body mass index, waist circumference, and steatosis) rather than microvascular complications.
- Pancreatic stiffness is not a reliable predictor of diabetic nephropathy or neuropathy.

## Introduction

The prevalence of diabetes mellitus (DM) and its complications represents a global health concern. According to the International Diabetes Federation, 1 in 11 individuals aged 20 to 79 had DM in 2015.^1^ It is estimated that this number will increase to 642 million by 2040. DM adversely affects the lives of patients with acute and chronic complications, including retinal, renal, neural, cardiac, and vascular complications.^2^

The pancreas consists of closely related endocrine and exocrine components, and alterations in 1 component may influence the other. Previous studies have reported increased pancreatic tissue stiffness in patients with DM, with potential associations with diabetic complications.^3^ 2D-shear wave elastography (2D-SWE) enables measurement of the pancreatic stiffness (elasticity) using ultrasound.^4^ However, studies evaluating pancreatic stiffness in patients with type 2 DM (T2DM) are limited, and its association with diabetic microvascular complications remains unclear.

In this study, the authors aimed to evaluate pancreatic stiffness using ultrasonographic 2D-SWE in patients with T2DM and to investigate its association with diabetic microvascular complications.

## Materials and Methods

This prospective case–control study was approved by the Mersin University Ethics Committee on October 14, 2020 (approval number: 704). This article was derived from the first author’s specialist thesis. The study was conducted between November 2020 and March 1, 2022. Patients with T2DM and healthy controls underwent detailed history taking, physical examination, laboratory evaluation, and comprehensive abdominal ultrasonography, including pancreatic 2D-SWE. The primary outcome was pancreatic stiffness measured by 2D-SWE. This pilot study aimed to evaluate the feasibility of pancreatic elastography measurements and provide preliminary data on pancreatic stiffness in patients with T2DM. Therefore, no predefined secondary endpoints were included. All participants were informed of the study purpose and scope, and written informed consent was obtained. Artificial intelligence (AI) was not used in the preparation of this manuscript.

### Inclusion Criteria

The patient group included individuals aged 18 to 80 years, who agreed to participate in the study and had a diagnosis of T2DM. The control group included healthy adults aged 18 to 80 years who presented to the outpatient clinic.

### Exclusion Criteria

Exclusion criteria included impaired glucose tolerance, acute and chronic hepatitis, acute and chronic pancreatitis, pancreatic cancer, pancreatobiliary surgery, being outside the 18- to 80-year age range, being pregnant or breastfeeding, having a body mass index (BMI) >35, alcohol use disorder (defined as consumption of >280 g of alcohol per week for men and >140 g per week for women), and a diagnosis of cancer.

### Clinical and Biochemical Parameters

For patients and controls, age at admission, comorbidities, and alcohol and smoking status were recorded. Anthropometric measurements such as height, weight, waist circumference (WC), and BMI were also obtained. After a 12-hour overnight fast, fasting blood samples were collected to measure fasting blood glucose, HbA1c, insulin, vitamin D, low-density lipoprotein, high-density lipoprotein (HDL), triglycerides, aspartate aminotransferase, alanine aminotransferase (ALT), alkaline phosphatase, gamma-glutamyl transferase, total bilirubin, albumin, urea, creatinine, and estimated glomerular filtration rate (eGFR). Postprandial blood glucose was measured 2 hours after a meal. WC was measured at the end of expiration, at the midpoint between the upper border of the iliac crest and the lower border of the 12th rib. BMI was calculated as weight in kilograms divided by height in meters squared (kg/m^2^). Insulin resistance was assessed using the homeostasis model assessment of insulin resistance (HOMA-IR) (fasting insulin (mU/mL) × fasting plasma glucose (mg/dL)/405). Individuals with a HOMA-IR of 2.5 or higher were classified as insulin resistant, whereas those with a HOMA-IR of 2.5 or less were classified as having normal insulin sensitivity. In the T2DM group, duration of diabetes (time since diagnosis), glycemic control, and the presence of diabetic nephropathy, diabetic neuropathy, and diabetic retinopathy were recorded. Microvascular complications were assessed prospectively. Retinopathy was diagnosed and evaluated by an ophthalmologist using indirect ophthalmoscopy to examine the fundus after dilated pupils. For the diagnosis of neuropathy, hot-cold and pinprick testing were performed to assess small-fiber function; the ankle reflex was assessed to evaluate large-fiber function; and vibration perception (using a 128-Hz tuning fork), 10 g monofilament test, and proprioception were evaluated. Diabetic nephropathy was defined based on serum creatinine level, eGFR, and microalbuminuria. High urinary albumin excretion was defined as a urine albumin/creatinine ratio of 30 mg/g or higher.^5,6^ The control group and patients with diabetes with and without microvascular complications (nephropathy, retinopathy, and neuropathy) were compared.

### Ultrasonographic Examination

Transabdominal ultrasonography (TAUS) was performed using a high-resolution ultrasound system (Toshiba Aplio 500, Tokyo, Japan) equipped with a 1- to 6-MHz convex transducer. Examinations were performed after a 12-hour fast. The liver and pancreas were evaluated in detail in all participants. Pancreatic parenchymal characteristics were evaluated and recorded. The structure and location of the pancreatic head, body, and tail, as well as pancreatic size, echogenicity, shape, and contours, were recorded. Pancreatic echogenicity was assessed by comparison with the parenchyma of the left hepatic lobe. A longitudinal scan along the abdominal midline was used to assess the echogenicity of the pancreas and liver at the same depth. When increased hepatic echogenicity was observed, the renal cortex was used as a reference for comparison. Pancreatic steatosis (PS) was defined as increased pancreatic echogenicity compared with that of the liver or renal cortex. Because the pancreas and kidney cannot be visualized simultaneously in the same ultrasound window, the ultrasonographer assessed the differences in echogenicity between the liver and renal cortex and between the liver and pancreas to provide an objective comparison of pancreatic and renal echogenicity. Pancreatic echogenicity was then categorized according to its degree of increase. The grading system used in this study was adapted from the previously described grading systems of Sezgin et al.^7^ Accordingly, PS was graded from 0 to 3.

### Two-Dimensional Shear Wave Elastography of Pancreas and Liver

During the ultrasound examination, elastography was performed. Patients were instructed to hold their breath, and once the image stabilized, the pancreas was clearly visualized without motion artifacts. This approach minimized movement-related artifacts as much as possible. A 10 × 10 mm region of interest (ROI) window was then positioned over the pancreatic tissue on the ultrasound monitor, avoiding the liver parenchyma and nearby arteries or digestive tract structures. After placement of the ROI, an SWE impulse was initiated. The depth of the ROI and pancreas stiffness (kPa) were displayed after a brief interval of approximately 1-2 seconds. Five measurements were obtained for each pancreatic segment, and the median of the 5 measurements was considered the valid value. Transverse or slightly oblique transverse sections were used.^7^^-^^9^ Liver stiffness (kPa) was measured from the right intercostal space. After ultrasonographic visualization of the right hepatic lobe, the ROI was placed 1-3 cm below the liver capsule, and an SWE impulse was applied. The median of 10 measurements (kPa) was recorded as the final liver stiffness value.

Pancreatic body stiffness was used for correlation analyses because previous internal analyses showed comparable stiffness values across the pancreatic head, body, and tail. Therefore, the pancreatic body was selected as the most reproducible representative segment.^7^

### Statistical Evaluation

In the statistical analyses, continuous variables were summarized as mean ± SD, and categorical variables were summarized as frequency and percentage. The normality of the data distribution was assessed using the Shapiro–Wilk and Kolmogorov–Smirnov tests. Comparisons between 2 groups were performed using the independent *t*-test, whereas analysis of variance was used for comparisons among more than 2 groups. The chi-square test was used for categorical variables. The Pearson correlation coefficient was used to assess linear relationships between variables. Linear regression analysis with stepwise variable selection method was used to further assess relationships between variables. Receiver operating characteristic (ROC) curve analysis was performed to evaluate the diagnostic performance of pancreatic stiffness in predicting diabetic microvascular complications. For all tests, statistical significance was set at *P* < .05. Statistical analyses were performed using Statistica 13. Based on an effect size of 0.63 units for BMI between patients with diabetes and controls,^3^ with a 5% type I error rate and 80% power,^10^ the minimum sample size required to detect a statistically significant difference between the groups was calculated as 45 individuals per group, for a total of 90 individuals. The sample size was calculated using the free version of the G-Power program version 3.1.9.7 (Heinrich-Heine-Univercity Düsseldorf; Düsseldorf, Germany).^11^

## Results

A total of 90 individuals were included in the study, comprising 45 controls and 45 patients with T2DM. Among the patients with T2DM, 19 had no microvascular complications and 26 had microvascular complications. A detailed description of the groups is provided in Table 1.

When the diabetes and control groups were compared (Table 1), statistically significant differences were observed in age, BMI, WC, fasting glucose, postprandial glucose, HbA1C, insulin, HDL, ALT, urea, creatinine, and GFR. Patients with DM, with or without microvascular complications, were also compared with one another and with the control group (Table 1).

Among the patients with diabetes and microvascular complications (n = 26), nephropathy was present in 11 patients (24.4%), retinopathy in 4 patients (8.9%), and neuropathy in 23 patients (51.1%). Regarding the number of complications, 17 patients (37.8%) had a single complication, whereas 6 (13.3%) and 3 (6.7%) patients had 2 and 3 complications, respectively.

### Relationship of Pancreatic Thickness Between Groups

The mean pancreatic head thickness was significantly greater in patients with T2DM than in controls (28.07 ± 6.24 mm vs 24.24 ± 7.69 mm, respectively; *P* = .026). No significant differences were observed in pancreatic body thickness (15.72 ± 3.17 mm vs 16.33 ± 4.47 mm, respectively; *P* = .515) or tail thickness (15.77 ± 2.99 mm vs 17.27 ± 3.69 mm, respectively; *P* = .073) between the T2DM and control groups. No statistically significant differences were observed in pancreatic head, body, or tail thickness among patients with T2DM with and without microvascular complications and controls (*P* = .081, .081, and .072, respectively).

### Pancreatic Tissue Stiffness in Patients with Type 2 Diabetes and Controls

Assessment of pancreatic tissue stiffness using 2D-SWE revealed a significant increase in stiffness across all anatomic regions in patients with T2DM compared with controls (*P* < .001 for head, body, and tail). The mean stiffness values in the T2DM group were 8.74 ± 1.66 kPa, 8.62 ± 1.98 kPa, and 8.74 ±1 .84 kPa for the head, body, and tail, respectively. In contrast, the control group had significantly lower pancreatic stiffness values, at 7.08 ± 1.43 kPa for the head, 6.99 ± 1.42 kPa for the body, and 6.89 ± 1.40 kPa for the tail (Table 2, Figure 1).

When the diabetes group was stratified according to the presence of microvascular complications, pancreatic stiffness values in both subgroups remained significantly higher than those in the control group. However, no statistically significant differences in stiffness were observed between patients with diabetes with and without microvascular complications across any pancreatic segment (*P* > .05). These findings, which demonstrate the diffuse and uniform nature of pancreatic structural changes in T2DM, are summarized in Table 2 and illustrated in Figure 1.

### Correlation of Pancreatic Body Tissue Stiffness with Biochemical and Clinical Data in Diabetic Patients

In the T2DM group, pancreatic body stiffness was positively correlated with age, BMI, WC, liver tissue stiffness, postprandial blood glucose, HbA1c, urea, and creatinine. In contrast, pancreatic body stiffness was negatively correlated with GFR (Table 3). The diagnostic performance of pancreatic stiffness in predicting microvascular complications was evaluated using ROC curve analysis. For diabetic nephropathy, sensitivity was high for the head (90.9%), body (100%), and tail (77.8%); however, specificity was low (29.4%, 23.5%, and 53.6%, respectively). The area under the curve (AUC) values for all pancreatic regions were approximately 0.55 and did not reach statistical significance (*P* > .05), indicating limited discriminatory ability. Consequently, no statistically significant cutoff values could be established (Figure 2). Similarly, for diabetic neuropathy, sensitivity was high (82.6%) across all pancreatic regions, whereas specificity was limited to 36%^9^. The AUC values ranged from 0.57 to 0.63 and did not demonstrate significant diagnostic discriminatory power (*P* > .05) (Figure 3).

### Regression Analysis of Waist Circumference for Pancreatic Body Stiffness

In regression analysis, WC explained 40% of the total variance in pancreatic body tissue stiffness, with statistical significance. For each 1-unit increase in WC, pancreatic body tissue stiffness increased by 0.112 units after adjustment for the other variables (*P* < .001) (Table 4). WC was identified as an independent determinant of pancreatic body stiffness in patients with T2DM.

In the analysis of covariance (ANCOVA) performed to account for the difference in age between the T2DM and control groups, age did not contribute significantly to the differences in elastography values between the groups for any pancreatic region (Table 5, *P* > .05).

### Pancreatic Steatosis and Pancreatic Tissue Stiffness

PS was present in 32 (71.1%) of 45 patients with T2DM and 16 (35.6%) controls (*P* = .001). Among patients with PS, 19 had mild, 11 had moderate, and 2 had severe PS. In patients with T2DM, pancreatic tissue stiffness was significantly higher in those with PS than in those without PS (9.01 ± 2.00 kPa vs. 7.65 ± 1.62 kPa, *P* = .035). No significant differences were observed in diabetes duration (*P* = .480), HbA1c levels (*P* = .191), or the presence of microvascular complications (*P* = .222) between patients with and without PS. Pancreatic tissue stiffness increased progressively with increasing severity of PS. Body elastography values were 8.61 ± 1.99 kPa for mild PS, 9.22 ± 1.85 kPa for moderate PS, and 11.80 ± 0.28 kPa for severe PS (*P* = .021 for the overall comparison among PS severity groups).

### Multivariate Analysis of Age, Body Mass Index, and Pancreatic Steatosis for Pancreatic Body Stiffness

Despite the significant difference in age observed between the diabetes and control groups (Table 1), age was not significantly associated with pancreatic tissue stiffness in the multivariate analysis, although it was included in the model (Table 6, *P* = .783). In contrast, BMI (*B* = 0.17; 95% CI, 0.06, 0.29; *P* = .004) and the presence of PS (*B* = 0.72; 95% CI, 0.20, 1.24; *P* = .008) were independently and positively associated with pancreatic tissue stiffness. Based on the standardized coefficients, BMI was the strongest predictor in the model (*β* = 0.40), followed by PS (*β* = 0.37).

## Discussion

This study provides a comprehensive evaluation of pancreatic changes in patients with T2DM using advanced 2D-SWE. The findings demonstrate that T2DM is associated with increased pancreatic tissue stiffness and morphometric alterations, particularly reduced pancreatic head thickness. Importantly, the findings suggest that metabolic burden, characterized by central obesity and PS, is more strongly associated with increased pancreatic stiffness than microvascular complications, including nephropathy or neuropathy. To the best of the authors’ knowledge, this is among the few studies to quantify the association between visceral adiposity and pancreatic elasticity using a multivariate approach, highlighting distinct metabolic remodeling of the pancreas in patients with diabetes.

Type 2 diabetes mellitus is a chronic, progressive disease characterized by worsening hyperglycemia and increasing therapeutic resistance, primarily driven by a progressive decline in beta-cell function. Experimental models, such as the Goto-Kakizaki rat, have demonstrated that inflammation and extracellular matrix accumulation within and around the islets contribute to pancreatic islet fibrosis. In diabetes, hyperglycemia stimulates the proliferation of pancreatic stellate cells and promotes collagen secretion, leading to pancreatic fibrosis and mirroring the structural changes observed in chronic pancreatitis.^12^^,^^13^ Similar pathological processes have also been recognized in diabetic microangiopathy.^14^^,^^15^ The hallmark features of microangiopathy include basement membrane thickening and excessive production of extracellular matrix proteins in the arterioles of the retina, glomeruli, myocardium, skin, and muscle.^16^ This condition manifests clinically as diabetic nephropathy, neuropathy, and retinopathy. Based on these mechanisms, the authors hypothesized that pancreatic stiffness may be increased in patients with T2DM and further exacerbated in those with microvascular complications. Exocrine pancreatic insufficiency is common in patients with PS, during the early stages of acute pancreatitis, in chronic pancreatitis, and after pancreatic resection.^17^^,^^18^

In the evaluation of pancreatic morphometry, tissue thickness did not differ significantly among patients with DM, with or without microangiopathy, and the control group. Notably, pancreatic head thickness was reduced in the DM group compared with controls (24 vs 28 mm, *P* = .026), whereas thicknesses of the other pancreatic regions remained comparable among the groups. Previous imaging studies have demonstrated reduced pancreatic volume and parenchymal atrophy in patients with DM, suggesting that chronic metabolic injury may lead to structural remodeling of the pancreas.^19^ Although the reason for this finding is unclear, it may reflect regional anatomical heterogeneity of the pancreas or differences in susceptibility to diabetes-related structural changes. Alternatively, the limited sample size may have reduced the ability to detect subtle differences in the body and tail regions. Therefore, this finding should be interpreted cautiously and warrants confirmation in larger studies. Abdominal ultrasonography-based SWE has emerged as a reliable and effective modality for assessing pancreatic stiffness.^20^ Previous studies have consistently demonstrated increased pancreatic stiffness in patients with acute and chronic pancreatitis compared with healthy individuals.^21^^-^^23^ In acute pancreatitis, this increase has been attributed to inflammation, inflammatory cell infiltration, and interstitial edema, whereas in chronic pancreatitis it is primarily associated with progressive fibrotic remodeling of the pancreatic parenchyma. Furthermore, Sezgin et al^7^ reported significantly increased pancreatic stiffness in individuals with PS, with positive correlations between stiffness and both the degree of steatosis and the severity of metabolic syndrome. Consistent with these findings, the authors also observed a significantly higher prevalence of DM among patients with PS.

Limited data are available in the literature regarding pancreatic stiffness specifically in patients with T2DM. In the initial study by He et al.^3^ which included 115 patients with T2DM and 115 controls, pancreatic tissue stiffness was significantly higher in patients with diabetes. Although no differences in pancreatic head and tail stiffness were observed between patients with and without microvascular complications, stiffness of the pancreatic body was significantly higher in those with complications. In a subsequent study of 213 patients with T2DM, He et al^24^ reported increased stiffness of the pancreatic head, body, and tail in patients with microvascular complications compared with those without complications. In this study, tissue stiffness of the pancreatic head, body, and tail was significantly higher in patients with diabetes and in both diabetes subgroups than in controls. However, no significant differences were observed between patients with and without microvascular complications. The authors hypothesize that the lack of differentiation between these complication subgroups may be attributable to limited statistical power, which may have obscured subtle differences between the clinical subsets.

In this study, pancreatic body stiffness was positively correlated with age, BMI, WC, HbA1c, liver stiffness, postprandial glucose, urea, and creatinine levels and negatively correlated with GFR in patients with T2DM. Previous studies have similarly used pancreatic body measurements to investigate associations between pancreatic stiffness and various biochemical and clinical parameters. Because age is a well-established determinant of increased pancreatic stiffness,^25^^,^^26^ the authors performed an ANCOVA to account for the older age of the T2DM group. This study demonstrated that age did not significantly influence the differences in elastography values between the diabetes and control groups across any pancreatic region. In contrast, WC, which was significantly higher in the DM group, was identified as an independent predictor of pancreatic body stiffness in patients with T2DM. This finding underscores the profound impact of central adiposity on pancreatic structural remodeling beyond the effects of chronological aging.

Furthermore, significant correlations between pancreatic stiffness and renal function markers—namely positive correlations with urea and creatinine levels and a negative correlation with GFR—led the authors to hypothesize that pancreatic stiffness might serve as a surrogate marker of diabetic nephropathy. However, subsequent ROC curve analysis demonstrated that pancreatic stiffness did not have statistically significant diagnostic performance for identifying diabetic nephropathy. Consequently, although intrapancreatic changes were evident, pancreatic stiffness did not reliably predict diabetic nephropathy in this cohort. Similarly, pancreatic stiffness did not demonstrate statistically significant diagnostic performance for predicting diabetic neuropathy. These findings suggest that the metabolic remodeling captured by 2D-SWE reflects localized pancreatic damage and steatosis rather than the systemic microangiopathic processes underlying diabetic complications. Therefore, 2D-SWE does not appear to be a reliable method for detecting diabetic microvascular complications.

As an integral component of the pancreatic ultrasonographic examination, the authors also assessed PS. PS was significantly higher in the DM group than in the controls, occurring in in 71.1% of patients with T2DM and 35.6% of controls (*P* = .001). Most cases were mild to moderate in severity. Pancreatic stiffness was significantly higher in patients with diabetes with PS than in those without PS. No significant differences were observed in DM duration, HbA1c levels, or the presence of microvascular complications (*P* = .222) between patients with and without PS. Pancreatic tissue stiffness increased progressively with increasing severity of PS.

PS occurs when pancreatic fat accumulation exceeds 6% and can be detected rapidly and accurately using methods such as TAUS. PS may impair pancreatic exocrine and endocrine functions and contribute to insulin resistance. PS is associated with the development and frequency of T2DM.^7^^,^^27^^-^^29^ A recent multicenter population-based study of 1700 individuals reported that the prevalence of PS in Türkiye is approximately 70%.^30^ Studies investigating this association have demonstrated significantly higher pancreatic stiffness in patients with PS than in those without PS.^7^^,25^ Furthermore, Sezgin et al,^7^ in a study of 125 participants, reported that pancreatic stiffness increased with increasing PS grade.

The study had several limitations. First, its single-center design and the relatively small sample size, which was influenced by the coronavirus disease-2019 pandemic, may have limited the statistical power of subgroup analyses, particularly those involving microvascular complications. Therefore, these exploratory analyses should be interpreted with caution. Similarly, the ROC analyses were performed in a limited number of participants and should be considered hypothesis generating rather than definitive. Larger prospective studies are needed to further investigate the relationship between pancreatic stiffness and diabetic microvascular complications. Second, although a significant difference in age was observed between the diabetes and control groups, multivariate and covariance analyses confirmed that age did not independently affect pancreatic stiffness, whereas metabolic factors such as BMI and PS remained significant. Nevertheless, the absence of age- and BMI-matched control subjects may have introduced residual confounding, limiting the authors’ ability to fully distinguish the independent contributions of diabetes, obesity, and PS to pancreatic stiffness.

Finally, the lack of reproducibility assessments and evaluations of inflammatory marker represents another limitation. Despite these limitations, this study provides valuable prospective insight into the metabolic driven structural remodeling of the pancreas in T2DM.

In conclusion, pancreatic stiffness was significantly higher in patients with T2DM than in healthy controls. Although microvascular complications were not significantly associated with pancreatic stiffness, multivariate analysis identified BMI and PS as the primary independent determinants of this structural change. Two-dimensional SWE may reflect metabolic associated structural alterations of the pancreas; however, these findings require validation in longitudinal and histopathological studies.

## Figures and Tables

**Figure 1.**
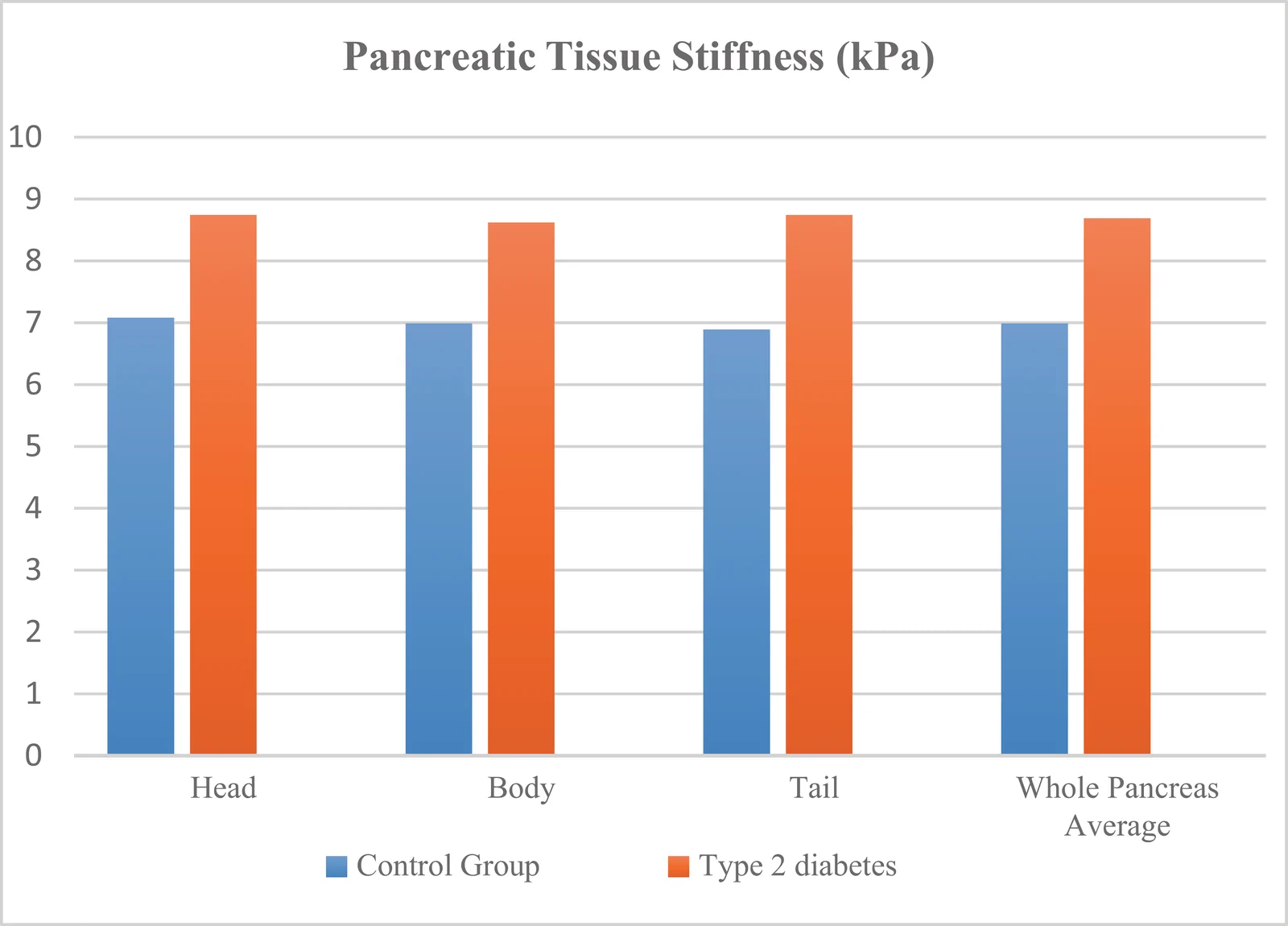
Pancreatic tissue stiffness in patients with diabetes and controls.

**Figure 2.**
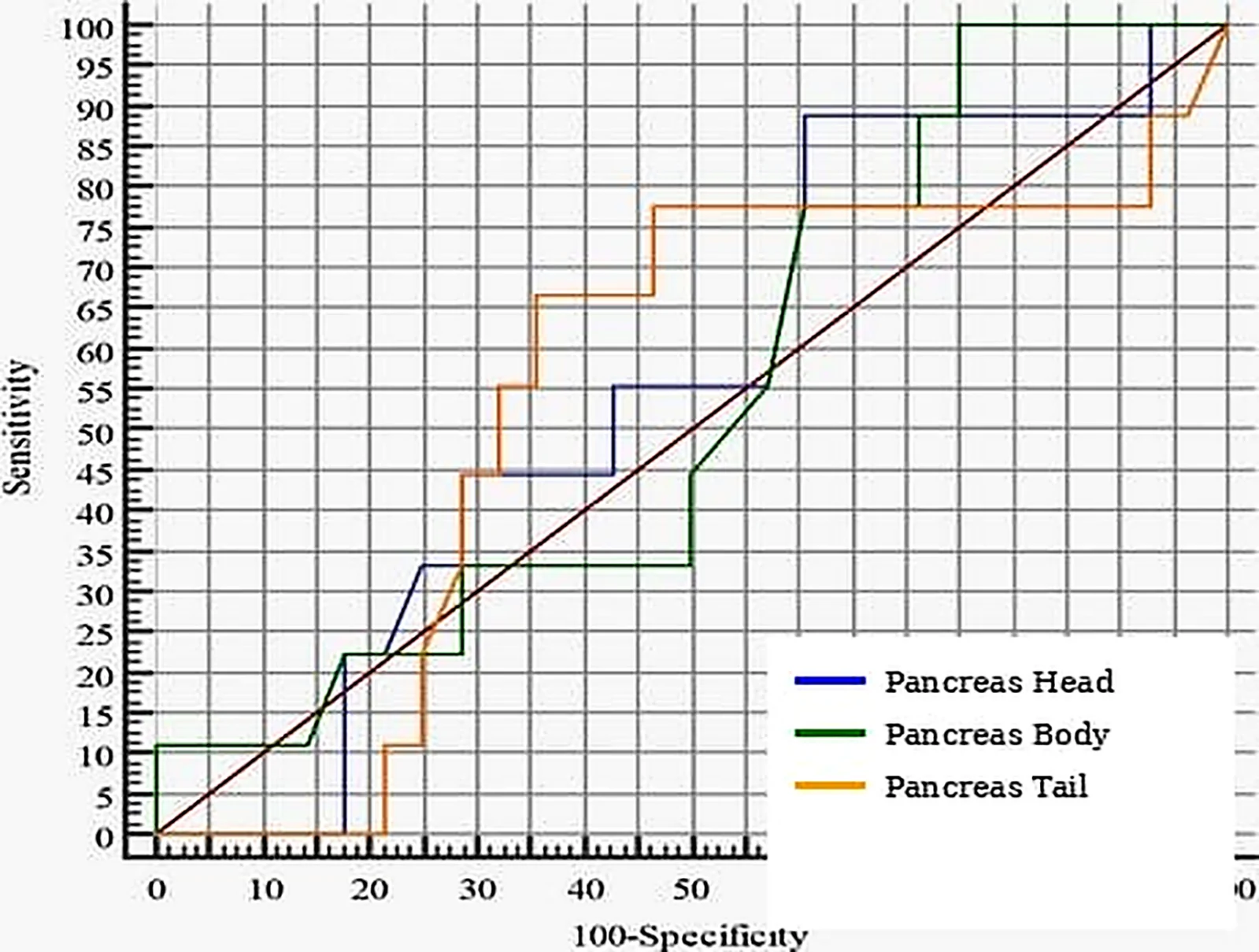
Diagnostic performance of pancreatic body elastography in patients with diabetic nephropathy.

**Figure 3.**
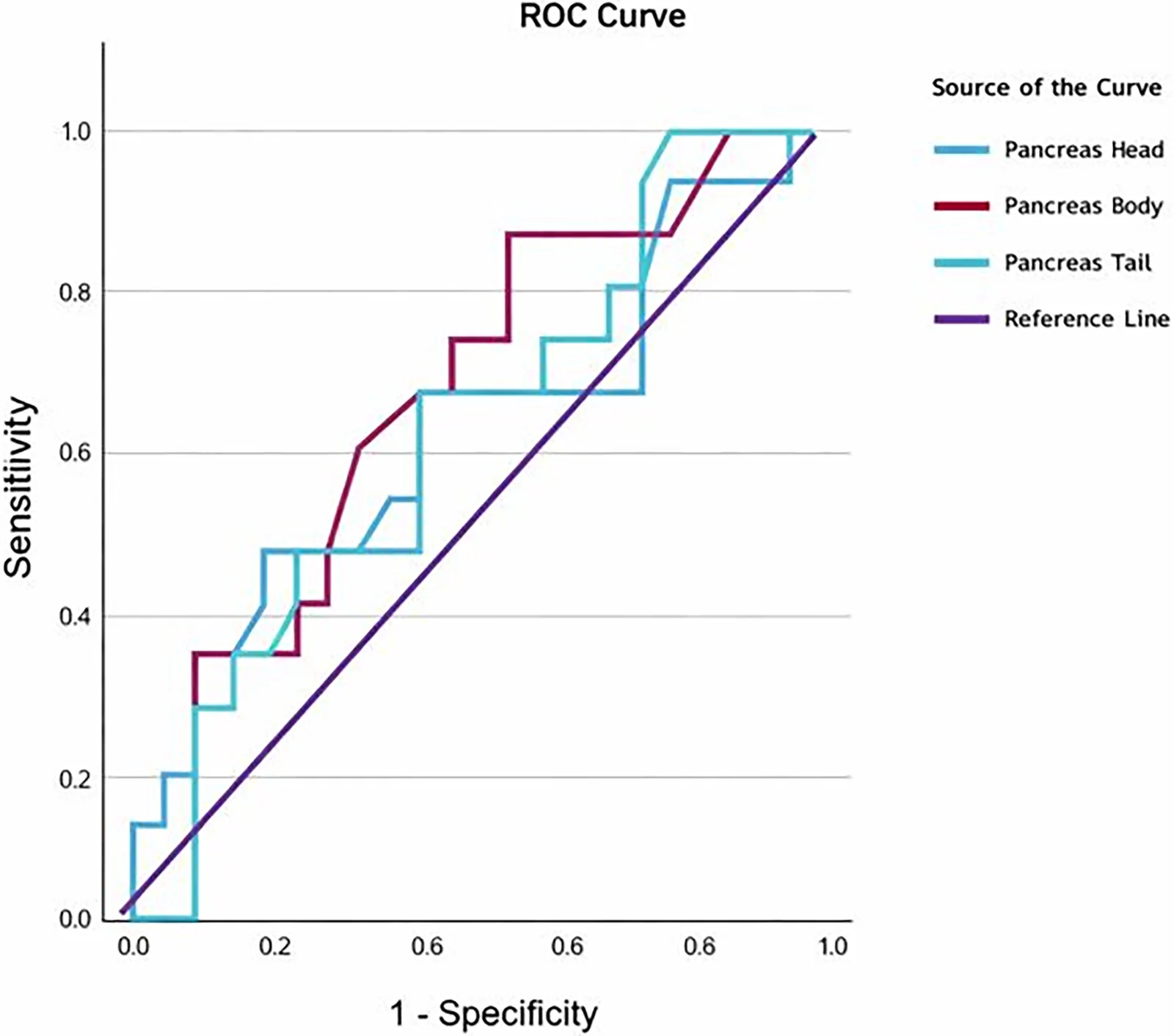
Diagnostic performance of pancreatic elastography in patients with diabetic neuropathy.

**Table 1.** Comparison of Biochemical and Clinical Data

Variables		Diabetics Without Microvascular Complications (n = 19)	Diabetics with Microvascular Complications (n = 26)	Control Group (n = 45)	Type 2 Diabetes Mellitus Patients (n = 45)	* P*
Gender (male/female)		13/6	13/13	15/30	26/19	
Age (years)		55.21 ± 11.54	57.88 ± 7.85	40.47 ± 14.49	56.76 ± 9.56	***P*** **1** **<** **.001**, ***P*** **2** **<** **.001**,*P*3 > .99, ***P*4 < .001**
DM duration (years)		5.68 ± 6.06	10.19 ± 7.14			***P*3 = .031 **
BMI (kg/m^2^)		28.45 ± 3.85	30.24 ± 3.75	24.51 ± 3.14	29.48 ± 3.86	***P*** **1** **<** **.001**, ***P*** **2** **<** **.001**,*P*3 = .273, ***P*4 < .001**
Waist circumference (cm)		97.63 ± 12.65	102.54 ± 11.04	82.76 ± 9.57	100.47 ± 11.87	*P* **1** **<** **.001**,*P* **2** **<** **.001**,*P*3 = .397, ***P*4 < .001**
Fasting glucose (mg/dL)		127.21 ± 37.54	155.92 ± 53.26	94.20 ± 8.24	143.80 ± 48.93	*P* **1** **<** **.001**,*P* **2** **=** **.002**,*P* **3** **=** **.018**, ***P*4 < .001**
Postprandial glucose (mg/dL)		171.11 ± 53.63	207.62 ± 91.41	99.38 ± 14.94	192.20 ± 79.10	*P* **1** **<** **.001** **,** *P* **2** **<** **.001**,*P*3 = .098, ***P*4 < .001**
HbA1c		6.81 ± 1.25	8.22 ± 2.49	5.37 ± 0.29	7.62 ± 2.16	*P* **1** **<** **.001**,*P* **2** **=** **.002**,*P* **3** **=** **.006**, ***P*4 < .001**
Insulin (µIU/mL)		11.03 ± 12.30	18.99 ± 28.68	6.12 ± 3.96	15.55 ± 23.20	*P* **1** **=** **.007**,*P*2 = .832, *P*3 = .344, ***P*4 = .009**
C-peptide (pmol/mL)		2.15 ± 0.49	3.34 ± 3.33	3.50 ± 0.67	3.00 ± 2.86	*P*1 > .99, *P*2 = .171, *P*3 = .370, *P*4 = .259
Vitamin-D (ng/mL)		32.84 ± 13.25	22.65 ± 10.17	28.40 ± 16.05	26.95 ± 12.51	*P*1 = .299, *P*2 = .749, *P*3 = .054, *P*4 = .636
Total cholesterol (mg/dL)		195.6 ± 35.03	185.57 ± 47.93	197.37 ± 47.84	189.71 ± 42.80	*P*1 > .99, *P*2 = .928, *P*3 > .99, *P*4 = .425
Triglyceride (mg/dL)		146.63 ± 103.95	165.23 ± 78.60	126.91 ± 70.73	157.38 ± 89.54	*P*1 = .173, *P*2 > .99, *P*3 > .99, *P*4 = .077
LDL (mg/dL)		121.00 ± 32.35	107.88 ± 40.29	120.45 ± 38.97	113.42 ± 37.33	*P*1 = .552, *P*2 > .99, *P*3 = .771, *P*4 = .385
HDL (mg/dL)		44.16 ± 10.56	43.35 ± 7.87	50.61 ± 10.44	43.69 ± 9.00	*P* **1** **=** **.01**,*P*2 = .055, *P*3 > .99, ***P*4 = .001**
AST (U/L)		28.47 ± 18.47	23.65 ± 10.63	21.62 ± 8.05	25.69 ± 14.47	*P*1 > .99, *P*2 = .103, *P*3 = .522, *P*4 = .103
ALT (U/L)		31.63 ± 22.40	25.26 ± 17.02	19.94 ± 12.18	27.95 ± 19.49	*P*1 = .556, *P* **2** **=** **.029**,*P*3 = .587, ***P*4 = .022**
ALP (U/L)		69.47 ± 14.49	70.62 ± 21.36	62.96 ± 17.94	70.13 ± 18.58	*P*1 = .282, *P*2 = .594, *P*3 > .99, *P*4 = .066
GGT (U/L)	27.00 ± 14.60		23.77 ± 14.03	20.47 ± 17.93	25.13 ± 14.20	*P*1 > .99, *P*2 = .434, *P*3 > .99, *P*4 = .175
Total bilirubin (mg/dL)	0.56 ± 0.17		0.60 ± 0.35	0.64 ± 0.25	0.59 ± 0.29	P1 = .611, *P*2 > .99, *P*3 = .718, *P*4 = .307
Albumin (g/dL)	4.45 ± 0.25		4.28 ± 0.33	4.35 ± 0.34	4.36 ± 0.31	*P*1 = .888, *P*2 > .99, *P*3 > .99, *P*4 = 949
Urea (mg/dL)	32.05 ± 8.32		35.61 ± 18.06	28.11 ± 7.93	34.11 ± 14.73	*P* **1** **=** **.029**,*P*2 = .576, *P*3 > .99, ***P*4 = .018**
Creatinine (mg/dL)	0.82 ± 0.17		0.88 ± 0.44	0.72 ± 0.19	0.85 ± 0.35	*P*1 = .093 *P*2 = .631 *P*3 > .99, ***P*4 = .032**
GFR (mL/dk)	92.79 ± 11.54		85.59 ± 23.34	107.96 ± 16.36	88.63 ± 19.41	*P* **1** **<** **.001**,*P* **2** **=** **.008**,*P*3 = .556, ***P*4 < .001**
Microalbuminuria (mg/g)	9.21 ± 7.04		101.75 ± 212.65			***P*3 = .002**

Data are presented as mean ± SD. ***P*1: **Control group versus diabetic patients with microvascular complications. ***P*2: **Control group versus diabetics without microvascular complications group. ***P*3**: Diabetic with and without microvascular complications. ***P*4**: Control group versus all of the diabetic patients. *P*-values reported as >.99 represent nonsignificant differences where the value was approximately 1.00 due to post-hoc adjustment. Bold values indicate statistically significant results (*P* < .05).

ALP, alkaline phosphatase; ALT, alanine aminotransferase; AST, aspartate aminotransferase; BMI, body mass index; DM, diabetes mellitus; GFR, glomerular filtration rate; GGT, gamma-glutamyl transferase; HDL, high-density lipoprotein; LDL, low-density lipoprotein.

**Table 2.** Pancreatic Tissue Stiffness in the Study Group

Variables	Diabetics Without Microvascular Complications (Mean ± SD)	Diabetics with Microvascular Complications (Mean ± SD)	Control Group (Mean ± SD)	Type 2 Diabetes (Mean ± SD)	*P*
Head elastography (kPa)	8.89 ± 1.83	8.63 ± 1.55	7.08 ± 1.43	8.74 ± 1.66	*P* **1** **<** **.001**,*P* **2** **<** **.001**, *P*3 > .99, ***P*4 < .001**
Body elastography (kPa)	9.08 ± 1.98	8.29 ± 1.96	6.99 ± 1.42	8.62 ± 1.98	*P* **1** **<** **.001**,*P* **2** **<** **.001**, *P*3 = .395, ***P*4 < .001**
Tail elastography (kPa)	9.11 ± 1.78	8.55 ± 1.88	6.89 ± 1.40	8.74 ± 1.84	*P* **1** **<** **.001**,*P* **2** **<** **.001**, *P*3 = .946, ***P*4 < .001**
Whole pancreas average (kPa)	8.98 ± 1.68	8.48 ± 1.65	6.99 ± 1.31		*P* **1** **<** **.001** **,** *P* **2** **<** **.001**, *P*3 = .805

***P*1**: Control group versus diabetic patients with microvascular complications. ***P*2**: Control group versus diabetics without microvascular complications group. ***P*3**: Diabetic with and without microvascular complications. ***P*4**: Control group versus all of the diabetic patients. Bold values indicate statistically significant results (*P* < .05).

**Table 3.** Correlation of Pancreatic Body Tissue Stiffness with Biochemical and Clinical Data in Diabetic Patients

Variables		Body Elastography (kPa)
Age (years)	*r*	0.332**
	*P*	**.001**
DM duration (years)	*r*	−0.147
	*P*	.336
BMI (kg/m^2^)	*r*	0.511**
	*P*	**<.001**
Waist circumference (cm)	*r*	0.517**
	*P*	**<.001**
Fasting glucose (mg/dL)	*r*	0.182
	*P*	.085
Postprandial glucose (mg/dL)	*r*	0.253*
	*P*	**.016**
HbA1c	*r*	**0.280** _**_
	*P*	**.007**
Urea (mg/dL)	*r*	0.222*
	*P*	**.036**
Creatinine (mg/dL)	*r*	0.211*
	*P*	**.046**
GFR (mL/min)	*r*	−0.313**
	*P*	**.003**
Number of microangiopathies	*r*	−0.201
	*P*	.186
Liver tissue stiffness (kPa)	*r*	0.378**
	*P*	**<.001**

*r*: correlation coefficient, *r* < 0.2, very weak; 0.2-0.4, weak; 0.4-0.6, moderate; 0.6-0.8, high; >0.8 very high. Bold values indicate statistically significant results (*P* < .05).

*P < .05, **P < .01.

BMI, body mass index; DM, diabetes mellitus; GFR, glomerular filtration rate.

**Table 4.** Waist Circumference Regression Analysis for Pancreatic Body Stiffness

Variables	*B*	*S*(*B*)	Beta	*t*	*P*
Constant	−3.033	2.712		−1.119	.274
Waist circumference (cm)	0.112	0.026	0.653	4.308	**<.001**

*R* = 0.65, *R*^2^ = 0.42, corrected *R*^2^ = 0.40 (*F* = 18.556; *P* < .001). Bold values indicate statistically significant results (*P* < .05).

**Table 5.** Statistical Contribution of Age to the Intergroup Variation of Pancreatic Tissue Stiffness

Region	The Source of Change	Squares Total	Degrees of Freedom	Squares Average	*F*	*P*
Head	Age	3.13	1.00	3.13	1.31	.260
	Groups patient control	31.02	1.00	31.02	12.99	< .001
Body	Age	3.93	1.00	3.93	1.32	.253
	Groups patient control	28.40	1.00	28.40	9.58	< .001
Tail	Age	2.745	1.00	2.745	1.053	.308
	Groups patient control	32.728	1.00	32.728	12.551	.001

**Table 6.** Multivariate Analysis of Age, Body Mass Index, and Pancreatic Steatosis for Pancreatic Tissue Stiffness

Variables	*B*	SE	*β*	*t*	*P*	95% CI
Constant	3.26	2.28	—	1.43	.161	[−1.35, 7.86]
Age (years)	−0.01	0.02	−0.04	−0.28	.783	[−0.05, 0.04]
BMI (kg/m^2^)	0.17	0.06	0.40	3.01	**.004**	[0.06, 0.29]
Pancreatic steatosis	0.72	0.26	0.37	2.80	**.008**	[0.20, 1.24]

*R*^2^ = 0.34, adjusted *R*^2^ = 0.30, *F* = 7.13, *P* < .001. Bold values indicate statistically significant results (*P* < .05).

BMI, body mass index; SE, standard error; *t,* *t*-statistic.

## Data Availability

The data that support the findings of this study are available on request from the corresponding author.
